# A novel approach to genome-wide association analysis identifies genetic associations with primary biliary cholangitis and primary sclerosing cholangitis in Polish patients

**DOI:** 10.1186/s12920-016-0239-9

**Published:** 2017-01-06

**Authors:** Agnieszka Paziewska, Andrzej Habior, Agnieszka Rogowska, Włodzimierz Zych, Krzysztof Goryca, Jakub Karczmarski, Michalina Dabrowska, Filip Ambrozkiewicz, Bozena Walewska-Zielecka, Marek Krawczyk, Halina Cichoz-Lach, Piotr Milkiewicz, Agnieszka Kowalik, Krzysztof Mucha, Joanna Raczynska, Joanna Musialik, Grzegorz Boryczka, Michal Wasilewicz, Irena Ciecko-Michalska, Malgorzata Ferenc, Maria Janiak, Alina Kanikowska, Rafal Stankiewicz, Marek Hartleb, Tomasz Mach, Marian Grzymislawski, Joanna Raszeja-Wyszomirska, Ewa Wunsch, Tomasz Bobinski, Michal Mikula, Jerzy Ostrowski

**Affiliations:** 1 Department of Gastroenterology, Hepatology and Oncology, Medical Center for Postgraduate Education, Warsaw, Poland; 2 Department of Genetics, Maria Sklodowska-Curie Memorial Cancer Center and Institute of Oncology, Roentgena 5, 02-781 Warsaw, Poland; 3 Department of Public Health, Faculty of Health Sciences, Medical University of Warsaw, Warsaw, Poland; 4 Department of General, Transplant and Liver Surgery, Medical University of Warsaw, Warsaw, Poland; 5 Department of Gastroenterology, Medical University, Lublin, Poland; 6 Department of General, Liver and Internal Medicine Unit, Transplant and Liver Surgery, Medical University of Warsaw, Warsaw, Poland; 7 Department of Immunology, Transplantology and Internal Medicine, Medical University of Warsaw, Warsaw, Poland; 8 Department of Gastroenterology and Hepatology, Medical University of Silesia, Katowice, Poland; 9 Department of Gastroenterology and Infectious Diseases, Collegium Medicum Jagiellonian University, Krakow, Poland; 10 Department of Gastroenterology, Provincial Hospital, Olsztyn, Poland; 11 Department of Gastroenterology and Hepatology, Medical University of Gdansk, Gdansk, Poland; 12 Department of Internal and Metabolic Diseases and Dietetics, Poznan University of Medical Sciences, Poznan, Poland; 13 Department of Clinical and Molecular Biochemistry, Pomeranian Medical University, Szczecin, Poland; 14 Department of Gastroenterology, Provincial Hospital, Ostroleka, Poland; 15 Institute of Biochemistry and Biophysics, Polish Academy of Sciences, Warsaw, Poland

**Keywords:** Genome-wide association study, Primary biliary cholangitis, Primary sclerosing cholangitis

## Abstract

**Background:**

Primary biliary cholangitis (PBC) and primary sclerosing cholangitis (PSC) are forms of hepatic autoimmunity, and risk for both diseases has a strong genetic component. This study aimed to define the genetic architecture of PBC and PSC within the Polish population.

**Methods:**

Subjects were 443 women with PBC, 120 patients with PSC, and 934 healthy controls recruited from Gastroenterology Departments in various Polish hospitals. Allelotyping employed a pooled-DNA sample-based genome-wide association study (GWAS) approach, using Illumina Human Omni2.5-Exome BeadChips and the following novel selection criteria for risk loci: blocks of at least 10 single nucleotide polymorphisms (SNPs) in strong linkage disequilibrium, where the distance between each adjacent SNP pair in the block was less than 30 kb, and each SNP was associated with disease at a significance level of *P* < 0.005. A selected index SNP from each block was validated using TaqMan SNP genotyping assays.

**Results:**

Nineteen and twenty-one SNPs were verified as associated with PBC and PSC, respectively, by individual genotyping; 19 (10/9, PBC/PSC) SNPs reached a stringent (corrected) significance threshold and a further 21 (9/12, PBC/PSC) reached a nominal level of significance (*P* < 0.05 with odds ratio (OR) > 1.2 or < 0.83), providing suggestive evidence of association. The SNPs mapped to seven (1p31.3, 3q13, 6p21, 7q32.1, 11q23.3, 17q12, 19q13.33) and one (6p21) chromosome region previously associated with PBC and PSC, respectively. The SNP, rs35730843, mapping to the *POLR2G* gene promoter (*P* = 1.2 × 10^-5^, OR = 0.39) demonstrated the highest effect size, and was protective for PBC, whereas for PSC respective SNPs were: rs13191240 in the intron of *ADGRB3* gene (*P* = 0.0095, OR = 0.2) and rs3822659 (*P* = 0.0051, OR = 0.236) along with rs9686714 (*P* = 0.00077, OR = 0.2), both located in the *WWC1* gene.

**Conclusions:**

Our cost-effective GWAS approach followed by individual genotyping confirmed several previously identified associations and discovered new susceptibility loci associated with PBC and/or PSC in Polish patients. However, further functional studies are warranted to understand the roles of these newly identified variants in the development of the two disorders.

**Electronic supplementary material:**

The online version of this article (doi:10.1186/s12920-016-0239-9) contains supplementary material, which is available to authorized users.

## Background

Primary biliary cholangitis (PBC) and primary sclerosing cholangitis (PSC) are hepatobiliary autoimmune diseases with strong genetic risk components [1]. PBC is characterized by progressive immune-mediated destruction of interlobular biliary ductules, and PSC by inflammatory and fibrotic processes affecting the intrahepatic and extrahepatic biliary tree; both diseases lead to liver cirrhosis [1–4]. PBC occurs more frequently in women than men and primarily in middle-age, with prevalences ranging from 40 to 400 patients per million and incidences ranging from 0.7 to 49 per million [5–8]. PSC affects 9 to 13 patients per million annually with a male-to-female ratio of 2:1 [3]. In Western countries, 60–80% of PSC cases are associated with inflammatory bowel disease (IBD), while PSC is present in 3–8% of all patients with ulcerative colitis and 1–3% of patients with Crohn’s disease [2, 9, 10]. Both PBC and PSC are classic polygenic disorders, with a genetic load represented by common, mostly non-protein-coding single nucleotide polymorphisms (SNPs), exhibiting similar small effect sizes [1].

Standard genome-wide association studies (GWASs) use high throughput SNP microarray technologies to discover SNPs associated with disease. Generally, the higher the sample size of a GWAS, the higher the number of the associations that reach the genome-wide significance threshold of *P* < 5 × 10^-8^ [2]. By contrast, studies with small sample sizes typically fail to reach GWAS statistical significance. The largest genetic association study for IBDs employed over 75,000 patients and controls and uncovered 163 IBD susceptibility loci [3]. However, studies of the heritable component of PBC and PSC by GWAS have been rather limited, primarily because of the lower prevalence of hepatobiliary autoimmunity, particularly PSC. While several GWASs have been performed using well-characterized PBC patient cohorts from North America, Europe, or Japan, only a few have included PSC patients mainly from northern Europe [4]; however, all of these GWASs had relatively small sample sizes [2]. Despite this, in addition to multiple PSC and PBC associations within the HLA complex, and with non-HLA genes mapping to the chromosome 6p21 major histocompatibility complex (MHC) locus, several associations have been identified at non-MHC susceptibility loci with genome-wide significance [6–17].

Many of these are associated with other immune-mediated diseases, including multiple sclerosis, celiac disease, and type 1 diabetes (T1D) [17, 18]. However, while larger GWAS cohorts, combining patients from different populations, may miss some sub-population-specific risk variants, smaller GWASs generally suffer from lack of statistical power. As a consequence, in GWASs limited to hundreds of patients, few or no associations at the accepted genome-wide significance level have been identified.

To identify susceptibility loci for PBC and PSC in the Polish population, we employed a pooled-DNA GWAS approach, together with a new method for identifying SNP associations. Considering the limited sizes of the cohorts investigated in our GWAS, this approach was effective in identifying novel PBC and/or PSC susceptibility loci. Despite the fact that “index SNPs” were selected at *P*-values much lower than the standard genome-wide significance threshold, the majority were validated by individual TaqMan SNP genotyping assays.

## Methods

### Studied subjects

Between 2010 and 2014, 443 women with PBC (389 antimitochondrial antibodies positive) and 120 PSC patients (37 females and 83 males) were recruited at nine recruitment centers. All enrolled patients and controls were Polish Caucasians. Diagnosis of PBC was based on standard clinical, biochemical, serological, and histological criteria, and PSC was diagnosed according to standard clinical, biochemical, cholangiographic, and in some patients - histological criteria according to the European Association for the Study of Liver (EASL) [19]. Blood samples were also obtained from 934 healthy individuals (512 females and 422 males). Sample sizes and age distribution for each group are shown in Table 1.

**Table 1:** Summary of the main epidemiological variables for the pooled-DNA allelotyping GWASs (A) and validation cohorts (B)

A.							
Type	Sex	Pooled-DNA GWAS					
		Enrolled (Set I)			Enrolled (Set II)		
		N^a^	Age, range (years)	Median age (years)	N^b^	Age, range (years)	Median age (years)
PSC	Females	40	12–61	31	37	17–52	31
	Males	80	9–64	26	74	9–49	26
	All	120	9–64	29	111	9–52	28
PBC	Females	420	14–85	53	407	29–80	53
	Males	0	0	0	0	0	0
		420	14–85	53	407	29–80	53
Healthy controls	Females	363	3–88	55	222	25–65	43
	Males	361	3–69	22	148	28–65	47
	All	724	3–88	50	370	25–65	44
B.							
Type	Sex	Individual patient genotyping					
		Enrolled					
		N	Age, range (years)		Median age (years)		
PSC	Females	41	17–52		31		
	Males	98	9–64		26		
	All	139	9–64		29		
PBC	Females	443	14–85		54		
	Males	0	0		0		
		443	14–85		54		
Healthy controls	Females	512	23–75		48		
	Males	422	27–78		48		
	All	934	23–78		47		

Panels indicate the number of patients enrolled in the GWAS (N^a^) after excluding microarrays that did not meet quality control criteria based on principal component analysis results (N^b^) that included validation and replication analyses performed using respective individual patient TaqMan genotyping (N). The range and median values are the ages of cases and controls in the respective groups in years

### GWAS allelotyping

A pooled-DNA sample-based GWAS was performed as described previously [20]. Genomic DNA was extracted from whole blood treated with EDTA using a QIAamp DNA Mini Kit. DNA sample concentrations were measured by a Quant-iTTM PicoGreen dsDNA Kit (Invitrogen, United Kingdom). DNA integrity was verified by 1% agarose gel analysis. Those DNA samples that passed quality control tests were combined according to diagnosis at equimolar concentrations to obtain two sets of DNA pools. The first microarray set consisted of 21, 6 and 30 DNA pools combining 20–24 DNA samples from PBC patients, PSC patients and healthy controls, respectively, whereas the second set consisted of 11, 3 and 10 DNA pools which were respectively combined from 37 DNA samples of PBC, PSC and controls. Pooled-DNA samples were adjusted to a final concentration of 50 ng/ml in Tris-EDTA buffer (pH = 8) and were assayed independently on Illumina Human Omni2.5-Exome BeadChips by the external AROS Applied Biotechnology A/S (Aarhus, Denmark) service.

### Individual genotyping

For validating the GWAS findings and the SNP typing replication study, individual patients and controls were genotyped with TaqMan SNP Genotyping Assays (Thermo, USA) using a TaqMan Universal Master Mix II (Thermo, USA) and a 7900HT Real-Time PCR system (Thermo, USA).

### Statistical analyses – allelotyping GWAS

For each SNP, on each microarray, the relative allele signal (RAS) was calculated as described previously [20]. RAS was used as an approximation of the allele ratio. The Student’s t-test (Welch variant) was used to compare allele ratios between groups. Due to a lack of the call-rate statistic for pooled samples, quality was assessed by visual inspection of first four principal components. A representative example is shown in Additional file 1: Figure S1. Six/two control pools were removed as outliers from the first/second microarray set, respectively. No probe filtering was performed. *P*-values were corrected for multiple hypothesis testing with the Bonferroni algorithm. Manhattan plotting was performed using the qqman R package [21]. All computations were performed according to R environment [22]. R: A language and environment for statistical computing. R Foundation for Statistical Computing, Vienna, Austria. URL http://www.R-project.org/).

### Statistical analyses – individual genotyping

Associations were examined using the Fisher-exact test implemented in R (version 3.1.1). The odds ratios (ORs) and 95% confidence intervals (CIs) were estimated by normal approximation using the EpiTools R package [23].

### Functional analyses

Symbols of genes to which SNPs mapped (in case of intergenic position symbols of both flanking genes) were used to assign functional overrepresentation using DAVID tool [24] and Swiss-Prot Protein Information Resource (SP-PIR) vocabulary.

## Results

For association screening, we adopted a cost-effective pooled-DNA GWAS design [20], which involved two separate hybridizations. Individual DNA samples from the same patient cohorts, that passed quality control, were combined in equimolar amounts, according to patient diagnosis, to obtain two sets of DNA pools from the same patients groups; the first set represented 420 PBC patients and 120 PSC patients and the second set, 407 and 111 of these two patient groups, respectively. In contrast, two different cohorts of 724 and 370 controls were employed. Selected SNPs, identified by GWAS, were genotyped using individual DNA samples from the same cohorts of 443 PBC patients, 120 PSC patients, and 934 control subjects in TaqMan SNP genotyping assays.

The method of selecting loci for validation is crucial for a successful GWAS. Our relatively small patient cohorts, particularly the PSC cohort, were statistically unpowered to detect SNPs associated at the standard threshold for genome-wide significance (*P* < 5 × 10^-8^). Therefore, assuming that an “index SNP” at a given locus is usually not independent of neighboring SNPs, we focused on loci forming blocks of at least 10 SNPs in strong allele linkage disequilibrium, defined by the following criteria: the distance between each pair of adjacent SNPs in the block was < 30 kb, and each SNP was associated with a disease at a significance level of *P* < 0.005. Among blocks meeting such criteria, we selected “index SNPs” associated with disease at *P* < 10^-4^, which were subsequently subjected to TaqMan SNP genotyping assays using individual PBC and PSC patient DNA samples. Although no extensive optimization of the number of required loci, *P*-value threshold, or window size was performed, the algorithm used was effective.

### Primary biliary cholangitis

Two rounds of GWAS led to the selection of 22 SNPs from 18 blocks of SNPs for validation as associated with PBC. Of the 22 SNPs, all but one were selected from the first GWAS, and only one of the seven SNPs selected from the second GWAS was not also identified in the first round. Among 22 “index SNPs” subjected to the genotyping assays, 19 were validated as associated with PBC, of which 10 reached the significance level determined by Bonferroni correction for multiple testing (*P* < 0.0023; 0.05/22), while the remaining nine demonstrated at least suggestive evidence for association, reaching a nominal level of significance (*P* < 0.05, with OR > 1.2 or < 0.83) (Additional file 2: Table S1). All 19 had the same direction of effect as observed in GWAS. In addition, two SNPs (rs3745516 and rs9303277) that were found to associate with PBC in previous studies [14, 16] were also confirmed to be associated in our PBC cohort. For 14 SNPs, the minor allele was associated with increased disease risk, while for the remaining five SNPs the minor allele showed a protective effect. The most significant PBC association (in terms of OR) as determined by genotyping assay was observed with the *POLR2G* locus (rs35730843; OR = 0.39; *P* = 1.2 × 10^-5^; SNP located in the gene promoter).

### Primary sclerosing cholangitis

Two rounds of GWAS led to selection of 29 SNPs originating from 22 blocks of SNPs for validation as associated with PSC; 18 and 12 SNPs were selected from the first and second GWAS rounds, respectively, and only one SNP was shared between both rounds. From the 29 SNPs, 21 were defined as associated with PSC after validation by genotyping assays, of which nine SNPs reached the level of significance determined by Bonferroni correction for multiple testing (*P* < 0.0017; 0.05/29) and 12 reached a nominal level of significance (*P* < 0.05 with OR > 1.2 or < 0.83) (Additional file 2: Table S2). Again, all 21 validated SNPs had the same effect direction as observed in GWAS. For 10 SNPs, the minor allele was associated with an increased risk, and for the other 11 the minor allele showed a protective effect. The most significant PSC association outside MHC region, as determined by genotyping assays, were observed at the *WWC1* gene (rs9686714; OR = 0.20; *P* = 0.00077; intronic location and rs3822659; OR = 0.24, *P* = 0.0051) and at the *ADGRB3* intron (rs13191240; OR = 0.2; *P* = 0.0095).

In addition, TaqMan SNP genotyping assays were used to test 18 and 3 SNPs associated with IBD and celiac disease, respectively, in a previous Polish study [25]. Of these, eleven and two SNPs were also found to associate with PBC and PSC, respectively, and another four SNPs with both PBC and PSC (Table 2). Finally, to assess which SNPs associated with PBC are shared loci for susceptibility to PSC and vice versa, all SNPs that had been subjected to validation in PBC were also genotyped in PSC, and those validated in PSC were genotyped in PBC. Altogether, 28 and 18 SNPs were unique to PBC and PSC, respectively, and eleven SNPs were shared between PBC and PSC. Of the latter, three had the same direction of effect in both types of hepatobiliary autoimmunity.

**Table 2:** Loci validated or replicated by individual patient TaqMan genotyping

Locus	SNP	Position	Location	Putative (nearby) gene(s)	PBC		PSC		MA, MAF
					*P*-value	OR	*P*-value	OR	
1p32	**rs4927257**	56046320	prom.		0.033	0.83	0.026	0.72	A, 0.439
2p25.3	rs1965732	3709108	intron	*ALLC*	0.028	1.24	0.029	1.41	G, 0.266
5q34	rs9686714	167853274	intron	*WWC1*	0.048	0.68	7.7E-4	0.195	C, 0.066
6p21	**rs2187668**	32605884	intron	*HLA-DQA1*	0.021	0.73	1.53E-7	2.47	T, 0.128
6p21	**rs9272346**	32604372	prom.	*HLA-DQA1*	0.0067	0.79	0.025	1.37	G, 0.422
6p21	rs7775228	32658079	inter.	*LOC100294145, C4B_2*	7.94E-9	1.84	0.00171	0.46	C, 0.146
6p21	rs3213489	32724305	intron	*HLA-DQB2*	8.37E-9	0.60	0.00155	1.56	T, 0.416
6p21	rs1799908	33144243	intron	*COL11A2*	0.032	1.21	0.0045	0.63	A, 0.405
6p21	rs7454108	32681483	inter.	*LOC100294145, C4B_2*	0.0090	1.43	0.00130	0.326	C, 0.086
9q34.3	rs10119096	139801932	intron	*TRAF2*	0.00087	0.70	0.041	0.70	T, 0.263
10q26.11	**rs17098094**	120592334	inter.	*MIR4681, CACUL1*	0.0174	0.81	0.032	0.73	G, 0.394
1p31.3	rs10489626	67793171	intron	*IL12RB2*	2.11E-5	1.59			G, 0.179
	rs3790567	67822377	intron	*IL12RB2*	8.4E-4	1.37			A, 0.242
1q32.1	**rs3024505**	206939904	inter.	*IL24, CTSE*	0.047	1.25			A, 0.158
2q31.1	rs2287619	169836730	intron	*ABCB11*	0.0079	1.50			C, 0.076
1q24.3	rs12118836	171256038	inter.	*MIR3120, MIR1295A*	0.0081	1.37			A, 0.145
3q13	**rs28413019**	109890863	inter.	*PVRL3-AS1, DPPA2*	0.022	1.25			G, 0.237
	**rs1491590**	70657915	inter.	*MIR1284, MITF*	0.0021	1.34			G, 0.251
4p15.1	rs13126571	27725689	inter.	*TBC1D19*	0.031	1.24			A, 0.230
4p14	rs3114381	40533297	intron	*RBM47*	0.0123	1.28			T, 0.232
4q26	rs979961	117850456	inter.	*TRAM1L1, MIR1973*	0.0062	1.39			C, 0.131
6p21	rs9268979	32435044	inter.	*LOC100294145, C4B_2*	6.73E-6	0.68			T, 0.471
6p21	rs3128927	33074288	inter.	*MIR1275, LOC100294145*	2.16E-5	1.50			T, 0.240
6q27	rs4710185	167522386	intron	*CCR6*	0.020	1.23			A, 0.358
6q27	rs9459874	167504127	intron	*CCR6*	0.0040	1.29			T, 0.425
6q27	**rs975822**	167516458	intron	*CCR6*	0.0042	1.29			T, 0.459
7q32.1	rs10488631	128594183	inter.	*SMKR1, LOC100130705*	0.0023	1.43			C, 0.132
7p14.3	rs965571	33622588	intron	*BBS9*	7.0E-4	1.43			C, 0.207
11q12.3	rs35730843	62527634	prom.	*POLR2G*	1.21E-5	0.393			C, 0.070
11p15.3	rs12786216	11275192	inter.	*MIR4299, MRVI1-AS1*	0.0076	0.77			C, 0.299
11q23.3	rs11217040	118680648	inter.	*LOC1001316*26	9.22E-5	0.66			A, 0.246
11q24.3	**rs73022813**	130704378	inter.	*SNX19, ADAMTS8*	0.043	1.24			A, 0.176
12p13.31	**rs7975557**	9853697	inter.	*KLRF2, MIR1244-1*	0.042	1.54			T, 0.035
14q21.1	**rs10083358**	39001998	inter.	*LOC100288846, MIPOL1*	0.0026	1.34			G, 0.252
16p12.3	**rs8055224**	20285401	inter.	*ERI2, IQCK*	0.0077	0.54			G, 0.050
16q23.3	rs8049648	83217488	intron	*CDH13*	0.00175	1.68			C, 0.061
19q13.33	**rs3745516**	50926742	intron	*SPIB*	7E-05	1.45			A, 0.254
21q22.11	**rs7279062**	32074387	inter.	*KRTAP21-3, KRTAP25-1*	0.0110	0.80			T, 0.476
17q12	**rs9303277**	37976469	intron	*IKZF3*	0.0051	1.27			T, 0.478
3q22.1	rs11917172	132166266	coding	*DNAJC13*			0.0122	0.53	G, 0.138
4p16.1	rs7686718	10171487	inter.	*CLNK, USP17L10*			0.00161	1.63	C, 0.261
6q12	rs13191240	69675459	intron	*ADGRB3*			0.0095	0.2	C, 0.04
5p15.31	rs6869702	6401135	inter.	*MIR4278, ICE1*			0.047	0.74	G, 0.409
5q22.2	rs1554624	111655382	intron	*EPB41L4A*			0.0126	0.55	G, 0.154
5q34	rs3822659	167858372	coding	*WWC1*			0.0051	0.24	TG, 0.053
6p21	**rs3130484**	31715882	intron	*MSH5-SAPCD1*			5.12E-11	3.23	C, 0.101
6p21	rs1264377	30764907	inter.	*PSORS1C3, MIR877*			8.02E-8	2.39	A, 0.159
6p21	rs2524163	31259579	intron	*HLA-B*			5.94E-7	2.02	C, 0.429
6p21	rs3130626	31598489	intron	*PRRC2A*			1.5E-6	2.10	G, 0.207
6p21	rs419788	31928799	intron	*SKIV2L*			1.21E-6	2.03	T, 0.250
6p22.1	rs3815081	30114074	intron	*TRIM40*			0.0141	0.59	G, 0.169
	rs1936365	28268452	intron	*PGBD1*			0.0034	1.72	C, 0.128
11q24.3	rs34708188	128223560	inter.	*SENCR*			0.0057	2.27	T, 0.038
7q35	rs954072	144757945	inter.	*CTAGE4*			0.0069	0.66	C, 0.406
8q21.13	rs1380634	80237893	inter.	*MIR5708, LOC101241902*			0.001011	1.70	G, 0.241
15q21.3	**rs11853454**	54169638	inter.	*UNC13C, WDR72*			0.040	1.39	A, 0.218
17p13.2	rs238224	4863410	intron	*SPAG7*			0.0174	0.57	A, 0.146

Map loci refer to the GRCh37 assembly. SNPs chosen on the basis of Celiac disease and IBD association are bolded. Odds ratios (ORs) are for minor alleles. *SNP* single nucleotide polymorphism, *MA* minor allele, *MAF* minor allele frequency; inter., intergenic (location); prom., promoter

Functional analysis of the merged validated SNP list for PBC and PSC revealed significant over-representation of genes associated with immune responses (*HLA-DQB1*, *HLA-DRB5*, *HLA-C*, *HLA-B*, *HLA-DPB1*, *HLA-DQA2*, *HLA-DQA1*, and *HLA-DRA*; *P* = 0.023; Additional file 2: Table S3).

## Discussion

GWASs provide information about common variants associated with disease susceptibility. Although GWASs allow the identification of disease risk alleles without prior knowledge of their position or biological function, they require large study cohorts to identify associations at the genome-wide significance threshold (5 × 10^-8^). By contrast, smaller GWASs using the same threshold may generate false-negative results. For association screening, we performed pooled-DNA GWASs and applied novel selection criteria that took into account strong allele linkage disequilibrium. We selected SNP blocks, defined by a distance of less than 30 kb between each pair of at least 10 SNPs associated with disease at *P* < 5 × 10^-3^, with an “index SNP” in the block for which the association was significant at *P* ≤ 10^-4^. All of the associations identified using this approach would have been missed using the standard genome-wide significance threshold. Of 22 and 29 associations with PBC and PCS, respectively, selected for validation, 19 and 21 SNPs were verified using TaqMan SNP genotyping assays of individual patient and control samples. In total, 19 SNPs reached the stringent (corrected) significance threshold, while the other 21 reached a nominal level of significance (*P* < 0.05 with OR > 1.2 or < 0.83), demonstrating at least suggestive evidence for association (Table 2). However, the expected number of false-positive results for 50 independent tests, with a significance threshold of 0.05, is < 3, while appropriate correction for multiple comparisons would reduce this to < 1. In our validation studies, correction for multiple testing reduced the number of associations at the nominal level of significance from 40 to 19, thus likely generating a large number of false-negative results and only slightly reducing the expected number of false-positive results.

This study identified 57 SNPs associated with either PBC or PSC or both disorders, which represent 38 genetic regions (Table 2). As expected, there were higher numbers of associations with HLA and non-HLA loci mapping to the MHC region (chromosome 6p21). Of 13 SNPs from this region, two, five, and six SNPs were associated with PBC, PSC, and both disorders, respectively. Of the SNPs shared between PBC and PSC, only two exhibited the same direction of effect.

As both PBC and PSC are hepatobiliary autoimmune diseases with low prevalence, the largest GWASs of these diseases have recruited individuals from different populations, which has certainly introduced a level of heterogeneity into the results, arising from the different genetic backgrounds of the geographically distinct populations. Consequently, these large cohort studies may have missed some subtle, sub-population-specific risk variants that may account for missing heritability. Conversely, studies with smaller sample sizes typically reveal a smaller fraction of the heritability of a complex disease, as they fail to detect associations because any found do not reach statistical significance thresholds [26]. The relative homogeneity of the Polish population may explain, at least in part, why our investigation identified so many SNPs significantly associated with PBC and/or PSC.

A GWAS including 536 North American PBC patients uncovered disease associations for several gene variants in the HLA class II region and coding variants in the interleukin-12a (*IL12A*) and IL12 receptor b2 (*IL12RB2*) genes [11]. Further GWASs have replicated these findings in a European population, and identified additional risk genes overlapping with other autoimmune diseases [17, 18]. In six GWASs, 27 non-HLA risk loci associated with PBC were identified [4]. Most have also been implicated in other autoimmune diseases, highlighting different immunoregulatory pathways. Our findings indicated the possible involvement of six previously described regions (1p31.3, rs3790567 [11]; 3q13 [13, 16]; 7q32.1, rs10488631 [12, 14]; 11q23.3 [13, 16]; 17q12, rs9303277 [18]; and 19q13.33, rs3745516 [14]) and the HLA-containing 6p21 locus in the development of PBC in Polish patients (Table 2).

Genomic studies of PSC have uncovered 18 associated genetic regions: 1p36 (*TNFRSF14*, *MMEL1*); 2q13 (*BCL2L1*); 2q33 (*CD28*); 2q35 (*GPBAR1*); 2q37.3 (*GPR35*); 3p21 (*USP4*, *MST1*); 4q27 (*IL2*, *IL21*); 6q15 (*BACH2*); 6p21 (HLA region); 10p15 (*IL2RA*); 11q23 (*SIK2*); 12q13 (*HDAC7*); 12q24 (*SH2B3*, *ATXN2*); 13q31 (*GPC5/6*); 18q21.1 (*TCF4*); 18q22 (*CD226*); 19q13 (*PRKD2*, *STRN4*); and 21q22 (*PSMG1*) [5, 6, 8, 27, 28]. Of these, only the MHC region was replicated in our PSC patients (Table 2).

The MHC region, which contains more than 224 genes and is highly polymorphic, is known to be associated with more than 100 different autoimmune and infectious diseases [29–31]. In European-based GWASs, the most pronounced MHC associations with PSC were with class I (*HLA-B* and *-C*) rather than class II (*HLA-DRB1* and *-DQB1*) loci [27]. A meta-analysis of three independent PBC cohorts identified HLA class II alleles (*HLA-DRB1*, *HLA-DQA1*, and *HLA-DQB1*) achieving genome-wide significance levels, with similar allele frequencies in Canadian, US, and Italian PBC cohorts [15]. Outside of the MHC region, our investigation confirmed six and zero genetic regions uncovered by previous GWASs as associated with PBC and PSC, respectively [27]. Of 30 chromosomal regions representing novel susceptibility loci, 13, 9, and 8 were associated with PBC, PSC, and both disorders, respectively. Of these, 17 SNPs have a shared genetic association with IBD, three with rheumatoid arthritis, two with lupus erythematosus, and single SNPs with psoriasis, lateral sclerosis, T1D, and intrahepatic cholestasis of pregnancy.

While well-designed GWASs should be conducted with groups of at least 1,000 patients and 1,000 controls, the appropriate level of statistical power to test for genetic associations (at *P* < 5 × 10^-8^) often relates to higher effect sizes [32]. However, since loci with a high effect size have generally been efficiently removed from the human population by natural selection, the identification of a common polymorphic susceptibility locus strongly associated with disease, with an OR > 2 or < 0.5, is unlikely [33]. Instead, a large number of previously identified loci associated with different disorders exhibit relatively small effect sizes, with ORs < 1.3. The present study uncovered only one SNP (rs35730843, *POLR2G*, *P* = 1.2 × 10^-5^, OR = 0.393) strongly associated with PBC and 11 SNPs strongly associated with PSC (rs3822659, coding in *WWC1*, *P* = 0.0051, OR = 0.236; rs9686714, intron of *WWC1*, *P* = 0.00077, OR = 0.195; rs13191240, intron of *ADGRB3*, *P* = 0.0095, OR = 0.2; rs7454108, intergenic between *LOC100294145* and *C4B_2*, *P* = 0.0013, OR = 0.326; rs2524163, intron of *HLA-B*, *P* = 5.9 × 10^-7^, OD = 2.02; rs2187668, intron of *HLA-DQA1*, *P* = 1.5 × 10^-7^, OR = 2.47; rs3130484, intron of *MSH5-SAPCD1*, *P* = 5.1 × 10^-11^, OR = 3.23; rs1264377, intergenic between *PSORS1C3* − *MIR877*, *P* = 8 × 10^-8^, OR = 2.39; rs3130626, intron of *PRRC2A*, *P* = 1.5 × 10^-6^, OR = 2.10; rs419788, intron of *SKIV2L*, *P* = 1.2 × 10^-6^, OR = 2.03; and rs34708188, intergenic close to *SENCR*, *P* = 0.0056, OR = 2.27). Of these, nine SNPs map to the MHC region (6p21), while *POLR2G*, *WWC1*, and *ADGRB3* are located in other genomic regions.

Our results indicated that a rare variant in the *POLR2G* gene promoter is associated with decreased risk of PBC, with a high effect size in the Polish population. *POLR2G* encodes one of the subunits in the polymerase 2 RNA complex, which is responsible for transcribing protein coding genes, miRNAs, and some classes of non-coding RNAs [34] and maps to the 11q12.3 locus, within which variants associated with chronic obstructive pulmonary disease [35] and asthma [36] have been identified. We also identified a decreased risk for PCS (OR < 0.25) conferred by a single rare variant in the *ADGRB3* gene and two rare variants (rs3822659 and rs9686714) in the *WWC1* gene. *ADGRB3* encodes transmembrane adhesion G protein-coupled receptor B3 (BAI3), which is broadly expressed in the brain and involved in the regulation of excitatory synapse connectivity [37]. Furthermore, BAI3 can promote myoblast fusion in vertebrates [38]. A previous GWAS identified SNPs at the *ADGRB3* locus as associated with early-onset venous thromboembolism [39]. *WWC1* encodes the KIBRA protein that plays versatile roles including in the regulation of cellular signaling, cell polarity, vesicular trafficking, and cell migration and division [40]. Specifically, KIBRA is a regulator of the Hippo signaling pathway, which controls tissue growth and tumorigenesis by inhibiting cell proliferation and promoting apoptosis [41]. Notably, *WWC1* hypermethylation occurs in 70% of B-cell acute lymphocytic leukemias [42] and its epigenetic silencing is also associated with unfavorable prognostic parameters in chronic lymphocytic leukemia [43]. Interestingly, the triggering of IL-6 trans-signaling, a process of aggregation of extracellular soluble IL-6 receptor and IL-6 associated with rheumatoid arthritis [44] and IBD [45], significantly increased *WWC1* expression in human airway smooth muscle cells [46], suggesting a link between its expression and inflammatory diseases. Importantly, rs3822659 is a missense variant (Ser735Ala) that alters the interaction of KIBRA with phosphatidylinositol 3-phosphate [47]. Other GWASs have implicated SNPs in *WWC1* as associated with memory performance and cognition [48], as well as Alzheimer’s disease [49].

## Conclusions

To the best of our knowledge, we have performed the first GWAS of PBC and PSC patients from the Polish population. Our cost-effective GWAS approach, followed by individual genotyping, allowed us to confirm several previously described associations and discover new susceptibility loci associated with both diseases. Although GWASs allow scanning of the entire genome to identify disease risk alleles without prior knowledge of their position or biological function, the use of statistical, rather than biological, criteria for selection of association findings greatly limits our understanding of the role of newly identified variants related to disease development. More importantly, the contribution of the genetic landscape in the context of environmental factors, and interactions between these two influences in conferring susceptibility to disease, has yet to be elucidated.

## Additional files

Additional file 1: Figure S1. Plot of first four principal components (PC) for: full dataset (A) and dataset after removal of outlier samples (B). Arrows indicate samples removed from analyses. PBC, primary biliary cholangitis; PSC, primary sclerosing cholangitis. (DOCX 298 kb)
Additional file 2: Table S1-S3.
**Table S1.** A summary of significant primary biliary cholangitis (PBC) loci selected based on GWAS results replicated by individual patient TaqMan genotyping. Position - position of a given loci on chromosome (GRCh37); GENESYMBOL - symbol of a gene SNP is located in, provided SNP is located within gene; PRECEDESYMBOL/FOLLOWSYMBOL - symbol of genes flanking given SNP, *p*-value - result of TagMan replication assessed with Fisher’s exact test; OR - odds ratio; PBC vs control (1st/2nd analysis) - indication whether given SNP met selection criterion in GWAS analysis, 1 = YES. Yellow marks SNPs significant in ‘PSC vs control’ comparison. **Table S2.** A summary of primary sclerosing cholangitis (PSC) loci selected based on GWAS results replicated by individual patient TaqMan genotyping. Position - position of a given loci on chromosome (GRCh37); GENESYMBOL - symbol of a gene SNP is located in, provided SNP is located within gene; PRECEDESYMBOL/FOLLOWSYMBOL - symbol of genes flanking given SNP, *p*-value - result of TaqMan replication assessed with Fisher’s exact test; OR - odds ratio; PSC vs control (1st/2nd analysis) - indication whether given SNP met selection criterion in GWAS analysis, 1 = YES. Yellow marks SNPs significant in ‘PBC vs control’ comparison. **Table S3.** Swiss-Prot Protein Information Resource Terms significantly overrepresented among genes associated with PBC and PSC in this study. (XLSX 21 kb)

## Abbreviations

GWAS: Genome-wide association study
IBD: Inflammatory bowel disease
MHC: Major histocompatibility complex
OR: Odds ratio
PBC: Primary biliary cholangitis
PSC: Primary sclerosing cholangitis
SNP: Single nucleotide polymorphism
