# Supplementary material for: A novel approach to genome-wide association analysis identifies genetic associations with primary biliary cholangitis and primary sclerosing cholangitis in Polish patients
Source: BMC Med Genomics. 2017 Jan 6;10:2. doi: 10.1186/s12920-016-0239-9 (PMC5217265; doi:10.1186/s12920-016-0239-9)
Supplement: Additional file 1: Figure S1. — Plot of first four principal components (PC) for: full dataset (A) and dataset after removal of outlier samples (B). Arrows indicate samples removed from analyses. PBC, primary biliary cholangitis; PSC, primary sclerosing cholangitis. (DOCX 298 kb) [file 12920_2016_239_MOESM1_ESM.docx]

**Additional file 1 - Figure S1.** Plot of first four principal components (PC) for: full dataset **(A)** and dataset after removal of outlier samples **(B)**. Arrows indicate samples removed from analyses. **PBC**, primary biliary cholangitis; **PSC**, primary sclerosing cholangitis
